# Oxygen absorption data of multilayer oxygen scavenger-polyester films with different layouts

**DOI:** 10.1016/j.dib.2018.06.024

**Published:** 2018-06-18

**Authors:** A. Apicella, P. Scarfato, L. Di Maio, L. Incarnato

**Affiliations:** Department of Industrial Engineering, University of Salerno, Via Giovanni Paolo II n. 132, 84084 Fisciano, SA, Italy

**Keywords:** Multilayer active film, Oxygen scavenger, Transport properties, Scavenging capacity, Exhaustion time

## Abstract

Oxygen absorption measurements in continuous regard active multilayer films with different layouts, all incorporating a PET/Oxygen scavenger (OS) layer, operating as active O_2_ barrier, inserted between two PET inert layers, acting as passive O_2_ barrier. The data set is related to “Transport properties of multilayer active PET films with different layers configuration” by Apicella et al. (2018) [1].

A set of four multilayer films, with different relative thickness of the active and inert layers, was produced using a laboratory scale co-extrusion cast-film equipment and was analyzed in terms of oxygen scavenging performance. Single layer active and inert layers were also produced for comparison. The results have shown a longer exhaustion time for all the active multilayer films, respect to the active monolayer one. Moreover, at constant thickness of the active layer, the exhaustion time increases by increasing the thickness of the inert layers, whereas, at constant thickness of the inert layers, the residual oxygen concentration decreases by increasing the thickness of the active layer.

**Table:** 

Subject area	*Engineering*
More specific subject area	*Material Technology*
Type of data	*Supplementary material*
How data was acquired	Optical oxygen meters Minisensor Oxygen Fibox 3-Trace V3 and Stand-alone Oxygen Meter Fibox 4 (PreSens GmbH, Regensburg, Germany)
Data format	*PDF graphs and tables, Excel 2016*
Experimental factors	*Active multilayer PET films with different layers configuration were produced using a laboratory scale co-extrusion cast-film equipment. The multilayer films incorporate a polyester/oxygen scavenger (OS) layer, operating as active O*_*2*_*barrier, inserted between two PET inert layers, acting as passive O*_*2*_*barrier. The polyester/OS layer was composed by PET (Cleartuf P60, M&G Polimeri) blended with a polyester-based oxygen scavenger (Amosorb DFC 4020, Colormatrix Europe) at 10% loading. Four different films layout were obtained, combining two thicknesses for the active layer and two for the inert layers.*
Experimental features	*The performances of the active multilayer films were related to the system layout.*
Data source location	*Department of Industrial Engineering, University of Salerno, Fisciano (SA), Italy*
Data accessibility	*Data are available with this article*

**Value of the data**

• In the literature, little experimental data focus on O_2_ transport and scavenging properties of multilayer active flexible systems
• The data demonstrate the key role of the multilayer film configuration in the film scavenging performance
• The experimental data allow to identify the best film configurations capable to maximize exhaustion times and scavenging capacity
• The provided data can be used for validation of diffusion/reaction mathematical models and parametric study of thickness configuration

## 1 Data

Experimental details are described in reference [1]. The data presented here are related to symmetrical, three-layer films (PET/PET+OS/PET) with different layouts, and single layer active (PET+OS) and inert (PET) films, as specified in Table 1. In these data, the individual contributions of active and inert layers on the films oxygen scavenging performance and properties were investigated.

**Table 1:** Nomenclature and description of the single layer and multilayer films produced with different layouts. Reprinted and modified with permission from [1].

Film nomenclature	Speed extruders [rpm]		Inert/active/Inert layer thicknesses [µm]	Total thickness [µm]
	A	I		
I	–	40	35/-/-	35
A	25	–	-/25/-	25
A_L_I_S_	40	27	6.75/23.5/6.75	37
A_S_I_S_	27	27	6.75/13.5/6.75	27
A_L_I_L_	40	40	11.75/23.5/11.75	47
A_S_I_L_	27	40	11.75/13.5/11.75	37

**Fig. 1:**
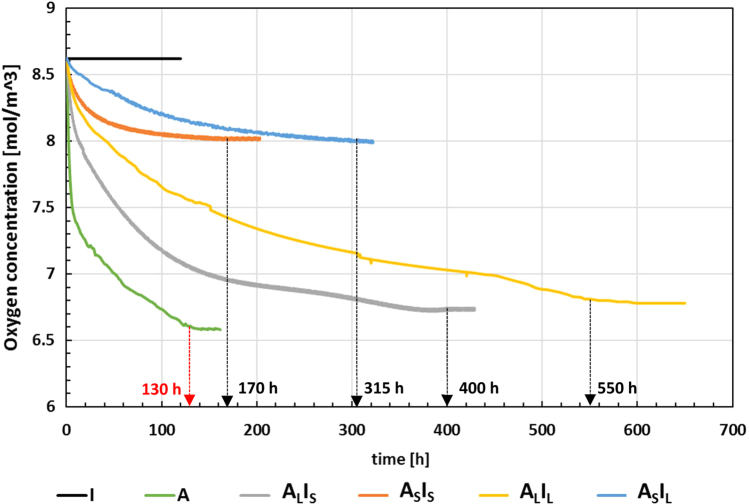
Oxygen absorption kinetics at 25°C for the single layer inert (I) and active (A) films and for the multilayer (A_L_I_S,_ A_S_I_S_, A_L_I_L_, A_S_I_L_) film samples, with different relative thicknesses. Reprinted and adapted with permission from [1].

The oxygen absorption kinetics for all the produced films are reported in Fig. 1, while the data points related to the curves are reported in the Excel file denoted as “Oxygen scavenging data”.

In addition, the resume of the oxygen absorption parameters calculated is shown in Table 2.

**Table 2:** Scavenging parameters of the produced single layer and multilayer films with different layouts. Reprinted and modified with permission from [1].

Sample	Initial O_2_ scavenging rate k	Exhaustion time t_E_ [h]	Residual O_2_ concentration in the vial [mol/m^3^]	Scavenging capacity µ_2_$\left[\frac{\mathrm{mL}O2}{µm\;\mathrm{active}\;\mathrm{layer}}\right]$
I	n.d.	n.d.	8.62	n.d.
A	0.257	130	6.58	0.0180
A_L_I_S_	0.094	400	6.73	0.0177
A_S_I_S_	0.086	170	8.02	0.0097
A_L_I_L_	0.041	550	6.75	0.0172
A_S_I_L_	0.046	315	7.99	0.0103

Fig. 1 shows no oxygen consumption for inert film I, whereas a decrease in oxygen concentration during the time (i.e. an oxygen consumption) is observable for all the active films.

With respect to the single layer active film A, all multilayer films show an increase of the exhaustion time. For multilayer films with the same thickness of the active layer (A_L_I_S_−A_L_I_L_ and A_S_I_S_−A_S_I_L_ pairs) the same increase of the thickness of the inert layer of 5 µm per side leads, in both cases, to the same increase in exhaustion time, of ≈ 150h (from 400 to 550 h and from 170 to 315 h respectively). The same film pairs also reach similar values of residual oxygen concentration in the vial (~6.7 mol/m^3^ for the A_L_I_S_−A_L_I_L_ pair and ~8 mol/m^3^ for the A_S_I_S_−A_S_I_L_ pair), which is lower for those films with larger thickness of the active layer.

**Fig. 2:**
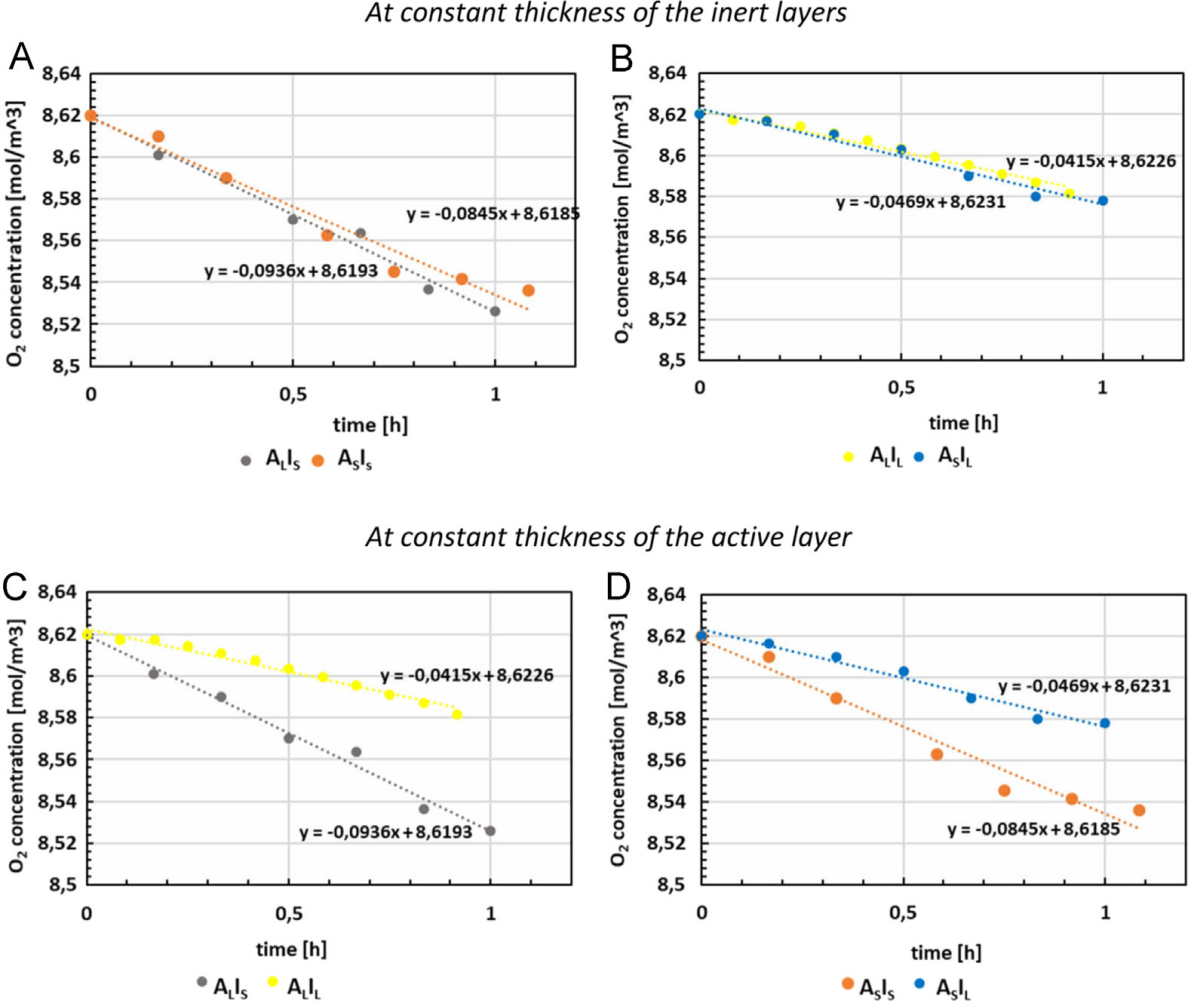
Comparison between initial oxygen scavenging rates k for multilayer films, at constant thickness of the inert layers ((A) and (B)) and at constant thickness of the active layer ((C) and (D)).

A comparison among the initial oxygen scavenging rates (i.e. the slopes of the oxygen absorption kinetics at short times) for the multilayer films is reported in Fig. 2. The values show a not significant variation for the samples with the same thickness of the inert layers (Fig. 2(A) and (B)). On the other side, at constant thickness of the active layer (Fig. 2(C) and (D)), the k value almost doubles by almost halving the thickness of the inert layers.

**Fig. 3:**
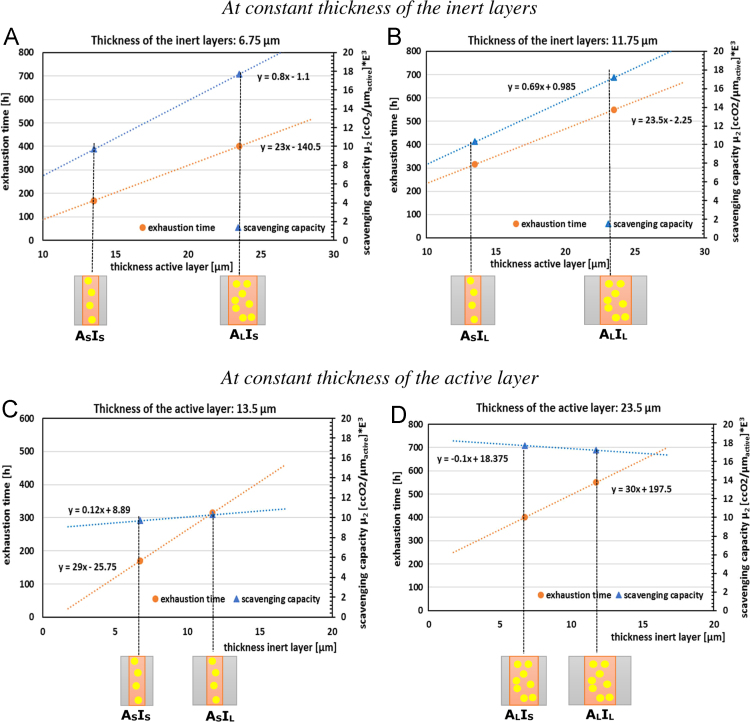
Dependence of exhaustion time and scavenging capacity µ_2_ on the multilayer layout; (A) and (B) on the thickness of the active layer, at constant thickness of the inert layers; (C) and (D): on the thickness of the inert layers, at constant thickness of the active layer. Reprinted and modified with permission from [1].

A comprehensive picture of the effects of the thickness of both the active and inert layers on the multilayers’ scavenging performance, in terms of exhaustion time and scavenging capacity µ_2_, is shown in Fig. 3. The graphs evidences that the coextruded active films with the same thickness of the inert layers (Fig. 3(A) and (B)) display an increase of both the exhaustion time and the scavenging capacity µ_2_ by increasing the thickness of the active layer, i.e. increasing the number of reactive sites available for the oxidation reaction. Moreover, similar increase trends are observable for both exhaustion time and scavenging capacity, as also observable from the slopes of the lines. On the other hand, the graphs related to the coextruded active films with the same thickness of the active layer (Fig. 3(C) and (D)) show similar scavenging capacity values for both pairs considered, while the exhaustion time increases, due to the increase of the external barrier layers, in similar way in both cases.

## 2 Experimental design, materials and methods

### 2.1 Materials

All materials were selected according to the results of our previous studies on coextruded and active films, and particularly on polyester/oxygen scavenger systems [1], [2], [3], [4], [5], [6], [7], [8], [9], [10], [11], [12], [13], [14], [15]. The selected polymeric matrix is the film grade PET resin Cleartuf P60 (M&G Polimeri S.p.A., Patrica (FR), Italy), having intrinsic viscosity 0.58 dL/g. The active phase is a new generation of polymeric oxygen scavenger, named Amosorb DFC 4020E (AMS, supplied by Colormatrix Europe, Liverpool, UK). This is a copolyester-based polymer designed for rigid PET containers, characterized by an auto-activated scavenging mechanism. Both PET and AMS comply fully with FDA and EU food contact legislation.

### 2.2 Multilayer film production

The PET was dried under vacuum at 130 °C for 16 h, before processing. The AMS, delivered dried in aluminum bags sealed under vacuum, was used as received. The percentage of the oxygen scavenger added to the active layer, equal to 10% wt/wt, was already optimized by previous studies [3].

The multilayer active films were produced by using a laboratory co-extrusion cast film line (Collin, Teach-line E20T), equipped with two single screw extruders (D = 20, L/D = 25) one feeding the active layer (A) and one feeding the inert layers (I), a flow convergence system (feed-block), a coat-hanger type head (slit die of 200 × 0.25 mm^2^) and a take-up/cooling system (chill rolls) thermally controlled by water circulation at 50 °C. The temperature profile for the two extruders was set at 280 °C from the hopper to the die. The chill roll speed was 7 m/min, thus allowing the films to be stretched to their final dimensions of about 170 mm wide and variable thicknesses.

The extruders were operated at different screw speeds, in order to modify the mass flow rate of the output materials and thus the relative layer thicknesses of the multilayer samples. Single layer films inert (i.e. made of pure PET) and active (i.e. made of PET + 10% wt/wt AMS) were also produced, for comparison, using the same apparatus and processing conditions.

### 2.3 Films characterization

Oxygen absorption measurements were carried out at 25 °C in continuous mode by means of the fiber optical oxygen meters Minisensor Oxygen Fibox 3-Trace V3 and Stand-alone Oxygen Meter Fibox 4 (PreSens GmbH, Regensburg, Germany), equipped with a polymer optical fiber and oxygen sensor spots SP-PSt3-NAU (detection limit 15 ppb, 0–100% oxygen). Experiments were conducted on cut film samples with a defined geometry (8 × 4.5 cm^2^), which were introduced in glass measurement cells, having volume equal to 9 ml, and hermetically capped. Then, oxygen consumption inside the closed glass vial was measured during the time. From the oxygen absorption curves, it was possible to calculate the residual oxygen concentration in the vial, the exhaustion time t_E_ (i.e. as the time at which the O_2_ concentration becomes constant), the initial oxygen scavenging rate k (i.e. the slope of the curves at short times, determined through a linear regression model by applying the ordinary least squares method to the experimental data of O_2_ concentration versus time). The scavenging capacity of the films at complete exhaustion was also evaluated, calculated as the ratio between the total volume of oxygen absorbed and the thickness of only the active layer (µ_2_).
